# Recombinant subunits of SARS‐CoV‐2 spike protein as vaccine candidates to elicit neutralizing antibodies

**DOI:** 10.1002/jcla.24328

**Published:** 2022-03-29

**Authors:** Faezeh Noorabad Ghahroodi, Saeed Khalili, Mohammad Javad Rasaee

**Affiliations:** ^1^ Department of Clinical Biochemistry Faculty of Medical Sciences Tarbiat Modares University Tehran Iran; ^2^ Department of Biology Sciences Shahid Rajaee Teacher Training University Tehran Iran

**Keywords:** in silico, neutralizing antibody, SARS‐CoV‐2, spike protein, vaccine

## Abstract

**Objectives:**

The spike protein has been reported as one of the most critical targets for vaccine design strategies against the SARS‐CoV‐2 infection. Hence, we have designed, produced, and evaluated the potential use of three truncated recombinant proteins derived from spike protein as vaccine candidates capable of neutralizing SARS‐CoV‐2 virus.

**Methods:**

In silico tools were used to design spike‐based subunit recombinant proteins (RBD (P_1_), fusion peptide (P_2_), and S1/S2 cleavage site (P_3_)). These proteins were checked for their ability to be identified by the anti‐SARS‐CoV‐2 antibodies by exposing them to COVID‐19 serum samples. The proteins were also injected into mice and rabbit, and the antibody titers were measured for 390 days to assess their neutralization efficiency.

**Results:**

The antibodies that existed in the serum of COVID‐19 patients were identified by designed proteins. The anti‐spike antibody titer was increased in the animals injected with recombinant proteins. The VNT results revealed that the produced antibodies could neutralize the cultured live virus.

**Conclusion:**

Truncated subunit vaccines could also be considered as robust tools for effective vaccination against COVID‐19. Using a combination of in silico, in vitro, and in vivo experiments, it was shown that the injection of spike‐based truncated recombinant proteins could stimulate long‐lasting and neutralizing antibody responses.

## Graphical abstract

Representation of corona vaccine development based on recombinant peptide following in‐silico studies.

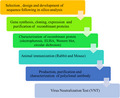

## 1 INTRODUCTION

The outbreak of the new coronavirus (SARS‐CoV‐2) is considered as the third viral disease of the coronavirus family in the 21st century.^1^ This virus rapidly spreads through the inhalation of respiratory aerosols. Clinical symptoms vary from no symptoms to highly significant clinical symptoms such as severe respiratory syndrome.^2^, ^3^ Primary transmitters of SARS‐CoV‐2 would be the patients with no signs and symptoms.^4^ Since the beginning of the COVID‐19 outbreak, many strategies and approaches have been proposed to fight against it. Disinfection of passages, quarantine, social isolation, public safety, drugs, and finally vaccines were the leading solutions offered by health care providers and specialists.^5^ Contemporary, various treatment strategies and vaccine production approaches are on the agenda all over the world. Oxygen therapy, antibiotics, antifungals, antivirals, glucocorticoids, and immunoglobulins are among the practiced treatment protocols.^2^, ^6^ Coming to grips with the life cycle and pathogenesis, mechanism of the SARS‐CoV‐2 would bring about insights into the proper strategies to develop vaccines.

SARS‐CoV‐2 is a single‐stranded RNA virus. It belongs to the Sarbecovirus subgroup of the Betacoronavirus group and the subfamily of the Orthomyxoviridae. Coronaviruses are divided into four groups including α, β, δ, and γ CoV.^7^, ^8^ The size of the viral genome is between 26 and 32 kb, which is considered as one of the largest RNA viruses.^9^ The diameter of these viruses is about 60–140 nm.^10^ Two‐thirds of the viral RNA transcribes for the pp1a, pp1ab, and 16 nonstructural proteins. The rest of the genome encodes for structural proteins.^11^ The main structural protein includes the spike (S), nucleocapsid (N), envelope (E), and membrane (M) proteins, which are encoded by the 3’ end of the viral genome.^11^, ^12^

SARS‐CoV‐2 enters the host cells through the angiotensin‐converting enzyme 2 (ACE2) receptor,^13^, ^14^ which resides in the human lower respiratory tract. It is also known as the cellular receptor for SARS‐CoV.^15^, ^16^ The S glycoprotein on the surface of the SARS‐CoV‐2 can bind to the ACE2 receptor on the surface of human cells.^17^ This protein is a trimmer protein that belongs to class 1 viral glycoproteins.^18^ It has been reported that the S proteins are the most important structural protein of the SARS‐CoV‐2 to enter the target cells. The S glycoprotein plays an essential role in viral binding, fusion, and entry into the host cell.^19^ This protein has two significant subunits known as S1 and S2 subunits. The S1 subunit identifies the host cell, while the S2 subunit is responsible for the fusion of the virus to the host cells.^20^, ^21^, ^22^ The N‐terminal domain (NTD) and the receptor‐binding domain (RBD) are the functional domains of the S1 subunit. The RBD encompasses a receptor‐binding motif (RBM) that is conserved among most strains of coronaviruses.^23^ The RBD sequence from the SARS‐CoV shares 72% similarity with the RBD from SARS‐CoV‐2.^24^, ^25^ Prior studies have revealed that the RBD can form a tight interaction with ACE2 protein, which initiates the infection process.^26^ The interaction between the SARS‐CoV‐2 and the ACE2 receptor mainly occurs between the RBM from the S protein and the N terminus region of the ACE2. This interaction leads to endocytosis of the virus.^26^ The interaction between the RBD and the ACE2 receptor stimulates structural changes in the S2 subunit. The exerted changes play an essential role in the fusion between the viral envelope and the host cell membrane. The S2 subunit of the S protein consists of several regions, including the membrane‐anchoring region, the fusion peptide (FP), the heptad repeat (HR) 1 and 2.^24^ Inside the endosome, the S1 subunit would be cleaved off and the FP would be exposed. The FP locates itself inside the host membrane. The S2 then bends over to bring HR1 and HR2 together. This causes membrane fusion and release of the viral genome in the cytoplasm of the host cell.^25^, ^27^, ^28^

According to WHO, tens of vaccines are under investigation worldwide at various stages. Various vaccine development strategies have been practiced in different settings preclinical and clinical trials, including the DNA, RNA, recombinant protein, viral vector, and the attenuated or deactivated viral vaccines. Safety considerations and the wide variety of antigen variants are potential challenges ahead of efficient vaccine development. The pivotal role of the S protein in the pathogenesis of the SARS‐CoV‐2 confirms that it can be the principal antigenic agent for stimulation of the host immune system and production of neutralizing antibodies.^26^ Prior studies have shown that vaccines made from the S protein can stimulate the immune system and induce humoral and cellular responses.^1^

The ongoing studies regarding the design of SARS‐CoV‐2 vaccines are already focused on the S protein using different platforms. In light of these observations, we aimed to design vaccine candidates based on the mechanism of S protein action and bioinformatics analyses. In this regard, three antigenic regions of the S protein were selected as vaccine candidates to elicit humoral immunization against SARS‐CoV‐2, which may induce neutralizing antibodies. The immunogenicity of these vaccine candidates was evaluated using in silico, in vitro, and in vivo studies.

## 2 METHODS

All procedures were performed according to the ethical guidelines of Faculty of Medical Sciences Tarbiat Modares University (TMU) and National Institute of Genetic Engineering and Biotechnology (code of ethics:1399.015).

### 2.1 Sequence retrieval

The sequence of the S protein was retrieved from the NCBI database at https://www.ncbi.nlm.nih.gov/. The obtained sequence was used to perform a BLAST search at https://blast.ncbi.nlm.nih.gov/Blast.cgi. The PSI‐BLAST (Position‐Specific Iterated BLAST) tool of the protein BLAST was employed to find the highly similar protein sequences. Multiple sequence analysis was performed on the sequences obtained from the BLAST search. The potential glycosylation sites on the S protein were predicted using NetNGlyc‐1.0 software at http://www.cbs.dtu.dk/services/NetNGlyc/. The glycosylation analyses would ensure the exclusion of glycosylated regions within the vaccine sequence. Since the prokaryotic expression system is unable to make accurate glycosylation on the produced antigens, the humoral responses against these regions would be rendered ineffective due to residing glycosylation on the spike protein. The S protein sequence was also searched for the existence of a signal peptide using SignalP‐5.0 software at http://www.cbs.dtu.dk/services/SignalP/.

### 2.2 Sequence analyses of the selected regions

The ProtParam software at https://web.expasy.org/protparam/ was used to predict the physicochemical properties of the selected regions. The potential glycosylation sites on the selected regions were predicted using NetNGlyc‐1.0 software. The antigenicity of the selected sequences was predicted by Vaxijen‐2.0 software at http://www.ddg‐pharmfac.net/vaxijen/VaxiJen/VaxiJen.html. The allergenicity of the regions was predicted by Algpred software at http://crdd.osdd.net/raghava/algpred/. The toxicity of the selected regions was predicted by ToxinPred software at http://crdd.osdd.net/raghava/toxinpred/.

### 2.3 Recombinant expression of the candidate vaccines

The protein sequence of the selected regions was reverse transcribed to the DNA sequences by the ExPASY translate tool at http://web.expasy.org/translate/. The Jcat tool at http://www.jcat.de/ was employed to optimize the DNA sequences for high levels of protein expression (the *E*. *coli* codon usage bias was used for the optimization). The EcoR1 and XhoI restriction sites were selected to insert the designed genes within the pET28a expression vector. This design would grantee the expression of His tag sequence at the N terminus of the proteins. The final genes were ordered for chemical synthesis and subsequent subcloning by the GENERAY Biotechnology Company. The synthesized genes (within the pET28a expression vector) were transformed into *E*. *coli* BL21 (DE3) using the standard CaCl_2_ method. Colony PCR using the universal T7 primers was employed to confirm the transformation. The protein expression of the transformed was performed using the same method employed in our previous study.^29^ The expression of the protein was optimized at different durations (4, 8, and 16 h), IPTG concentrations (0.3, 0.5, 0.8, and 1 mM), and temperatures (18, 25, and 37°C). The total proteins of the expressed cultures were analyzed by SDS‐PAGE in 15% (W/V) polyacrylamide gel. The gel was stained by standard Coomassie brilliant blue G‐250 for 4 h. De‐staining was carried out with 45% methanol and 10% acetic acid solution.

### 2.4 Protein purification and Western blotting

Protein purification and Western blot analysis were performed using the protocol adapted from our previous study.^29^ Briefly, the Ni^+^–NTA resin‐packed columns (Qiagen) and pH gradients were used to purify the expressed proteins following the procedures provided by the manufacturer. The SDS‐PAGE (4% stacking gel and 15% separating gel) was used to analyze the genes’ expression and the purity of the eluted fractions. The purification fraction (with the pH of 5.2), which contained a single protein band of the recombinant protein, was selected for the following experiments. The protein content of the eluted fractions was measured using standard Bradford assay. Since the proteins formed inclusion bodies, they were denatured using standard denaturing conditions by 8 M urea. The purified protein samples were finally dialyzed (for protein renaturation) to remove the urea and increase the pH from 5.2 to 7.4. A standard Western blot analysis was performed to assess the yield of the purification step. In this regard, primarily the proteins were resolved on the gel and was transferred onto the nitrocellulose membrane (Whatman Schleicher and Schuell). Ultimately, an anti‐His tag antibody conjugated with horseradish peroxidase (HRP) was added to the nitrocellulose membrane in order to visualize the reactive bands.^30^

### 2.5 Circular dichroism (CD) spectroscopy

Since the complete removal of the urea could lead to protein precipitation and aggregation, the urea was gradually removed by dialysis. CD Spectroscopy (Jasco's J‐810 spectropolarimeter) then assessed the effects of different urea concentrations on the secondary structure of the protein in the far UV absorption range (the wavelength range of 190–260 nm) (Far‐UV‐CD) of peptide bonds. Each secondary structure created a unique curve according to the rotation angle of its peptide bond. To perform the experiment, 0.2 mg/ml of each protein was dissolved in 10‐mM phosphate buffer for different molarities of urea and measured. The obtained spectra were corrected against PBS buffer and other molarities of urea base buffer (0, 1, 2, 4, 6, and 8) as a control using a unique noise reduction software. The data obtained in these experiments were analyzed at https://npsa‐prabi.ibcp.fr/ to calculate the percentage of secondary structures.

### 2.6 Animal immunization and production of polyclonal antibody

The study conduction was adhered to the principles of the declaration of ARRIVE guidelines based on Ethical Committee Compliance of TMU (code of ethics: 1399.015). Three groups (2 rabbits per each group) of female New Zealand white rabbits (8 weeks old) and four groups (3 mice per each group) of BALB/c female mice (4–6 weeks old) (Razi Institute, Karaj, Iran) were used to elicit polyclonal antibodies. Each group of rabbits received one of the candidate vaccine proteins, injected (intramuscularly into the large muscle of the rear legs) 350 μg along with the same volume of Freund's complete adjuvant. The booster injections were given at 15 day intervals with 350 µg of each vaccine candidate protein mixed with incomplete Freund's adjuvant. The first bleeding was performed before the injections. Bleedings were done after the third injection and repeated at 15‐day interval. The antibody titers of rabbit serum were measured using the indirect ELISA method. To determine the antibody titer, briefly, 1μg of the purified proteins was coated on a 96‐well microtiter plate at 37°C overnight, washed, and blocked with 5% skimmed milk. The coated wells were incubated with serially diluted serum (1:500, 1:1000, 1:2000, and 1:4000 in PBS). The wells were incubated with 1:4000 diluted HRP‐conjugated mouse anti‐rabbit IgG (Thermo Scientific) for 90 min at 37°C. Finally, 50 μl of tetramethylbenzidine substrate reagent (BD Biosciences) was added to each well and incubated for 15 min at 37°C. 50 μl of 2 N of HCl was used to stop the color development, and the absorbance was measured at 450 nm. In another sets of experiments, the mice were injected intramuscularly with 50 μg of each vaccine candidate protein along with the same volume of Freund's complete adjuvant. The booster injection was given twice after 15 days with 50 µg of each vaccine candidate protein mixed with incomplete Freund's adjuvant. The fourth mice group was injected with 150 μg of the three vaccine candidates (50 µg of each vaccine was mixed) mixed with incomplete Freund's adjuvant, and bleeding was performed every month. To determine the antibody titer, the indirect ELISA method was employed as explained before.

### 2.7 ELISA test on patient's serums

To assess the ability of the candidate vaccine proteins to interact with the antibody available in the serum of the patient (all study participants received a full explanation of the study and were obtained a written informed consent prior to their inclusion in the study), an ELISA test was designed and developed, and employed. Briefly, 1 μg of each purified protein was coated onto a 96‐well microtiter plates at 37°C overnight. Serum samples from 50 SARS‐CoV‐2 patients and healthy people were added to the wells, incubated and washed, 1/10,000 dilution of anti‐human IgG (Goat Anti‐Human immunoglobulin‐HRP conjugate) was added, incubated, and washed. 50 μl of tetramethylbenzidine substrate reagent (BD Biosciences) was added to each well, incubated for 15 min at 37°C, and added with 50 μl of 2 N of HCl to stop the color development.

Such type of assays was repeated for several samples obtained from people infected with the virus (already detected by RT‐PCR). In addition, a conventional rapid immuno‐chromatography test was used to observe the interaction of purified antibody with the antigen coated onto the membrane in such assays.

### 2.8 Antibody purification

The elicited antibodies should be purified for virus neutralization assay. Antibody purification for all serum samples was done using affinity chromatography on a Protein A Agarose column (PAO9‐R5, ABT Company). The purification was performed according to the manufacturer's instructions. Briefly, saturated ammonium sulfate was gradually added to rabbit sera at the final concentration of 33%. They were then stirred on ice for one hour. After 25 min of centrifugation at 9000 *g*, the supernatant was removed and led on the protein A column. The column was washed by PBS, and elution buffer (glycine‐HCl, pH = 2.5) was added to elute the desired protein, neutralized using carbonate buffer to adjust the pH, concentrated via Amicon ultrafiltration device cutoff concentrators (10 kDa), measured the protein content by standard Bradford protein assay, and was determined by SDS‐PAGE (4% stacking gel and 12.5% separating gel.

### 2.9 Preparation of cells and virus stock

To perform the neutralization assays, the African green monkey kidney, Vero E6 cells were employed. These cells were cultured in Dulbecco‐modified Eagle medium (DMEM) supplemented with 10% heat‐inactivated fetal bovine serum (FBS), 100 mg/ml of streptomycin, 100 units/ml of penicillin G, and 2‐mM L‐glutamine. The culture conditions were set to 5% CO_2_ at 37°C, and the cells were grown up to confluency of 70%–80%. The SARS‐CoV‐2 was obtained from COVID‐19 patients (strain hCoV‐19/Wuhan/WIV04/2019) and propagated in Vero E6 cells, adapting a method developed by Harcourt et al.^31^ A unique biosafety level 3 (BSL‐3) laboratory was used to handle the virus and the infected cell cultures. The procedures were performed following the instructions approved by Institutional Biosafety Committee (IBC).

### 2.10 Cytopathic effect (CPE) based neutralization assays

The CPE‐based neutralization assays were carried out in 96‐well microtiter plates in triplicate. Three rabbit sera (immunized by P_1_, P_2_, and P_3_ antigens), the serum‐free DMEM culture media (as a control for virus propagation), and the COVID‐19 virus stock (with ct of 12 (10^6^ TCID 50/ml)) were prepared in 1:10, 1:100, and 1:1000 dilutions. 250 µl of each viral dilution was then incubated with each dilution of the rabbit antibody for 2 h at 37°C. As the negative control group, 250 µl of each viral dilution was incubated for 2 h at 37°C with cell culture medium without any antibody. The antibody‐virus mixture was added on 20,000 Vero E6 cells (in 100 μl) at MOI of 0.02 and incubated for 2 h at 37°C. The incubation was continued up to CPE appearance, which was first observed on day 5 of infection under a microscope. The definition for neutralizing antibody titer was the reciprocal of the highest antibody dilution at which none of the triplicate testing wells was observed with CPE breakthrough.

## 3 RESULTS

### 3.1 Obtaining the proper sequences

The sequence of the S protein was stored under the reference Sequence ID of YP‐009724390.1. The BLAST search based on this sequence has returned numerous protein sequences with significant coverage and identity. The MSA results confirm the previous studies showing the conserved and variable regions of the S protein.^32^ The variability in the RBM sequence was also evidently analyzing the MSA results. There were 17 asparagine residues predicted to be N‐glycosylated throughout the S sequence. The glycosylation was less condensed at the RBD region and the region connecting the S1 and S2 parts. The sequence spanning the residues 1–15 was predicted to be a signal.

### 3.2 Selection of regions for vaccine candidates

Given the properties of the S protein and considering the mechanism of S protein to fuse the virus into the host cells, three areas, including the RBD (P_1_), fusion peptide (P_2_), and S1/S2 cleavage site (P_3_) of the spike protein, were selected to be the candidate vaccine antigens. Their molecular weight was calculated to be 11,477.82 Da, 13,528.71 Da, and 25,000 Da for P_1_, P_2_, and P_3_ antigens, respectively. The selected vaccine candidates were all predicted to be stable according to their instability index and have a high estimated half‐life within mammalian cells. Analyzing the properties of these antigens, there were no asparagine residues with possible glycosylation, all three vaccine candidates were predicted to be antigens, no allergenicity effects were expected to be inclined by these vaccine candidates, and there were no regions with significant potential of toxicity throughout their sequences.

### 3.3 Protein expression

**FIGURE 1:**
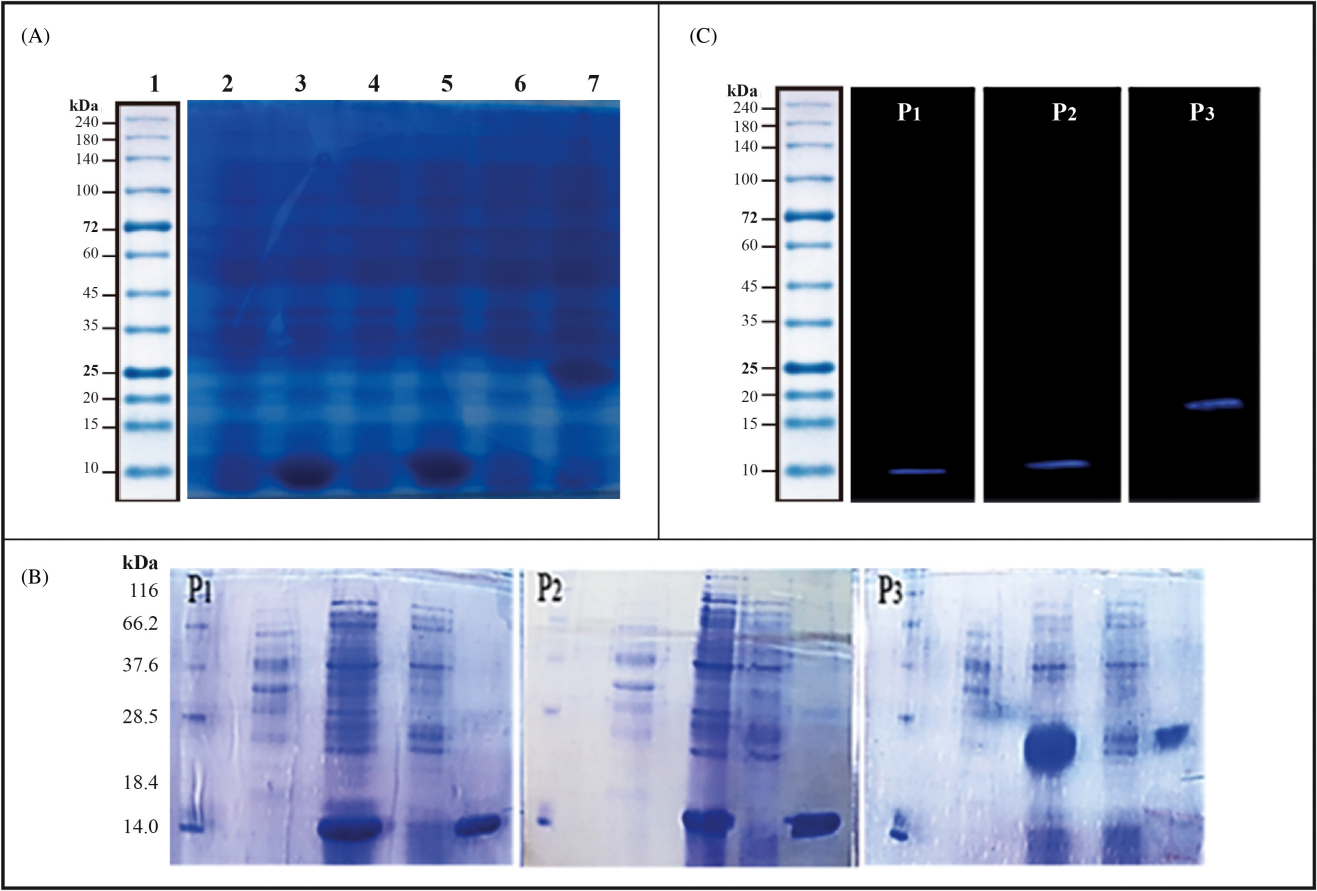
(A) SDS‐PAGE analyzes the protein expression (Lane 1. Protein ladder, Lane 2. P_1_ before expression, Lane 3. P_1_ after expression, Lane 4. P_2_ before expression, Lane 5. P_2_ after expression, Lane 6. P_3_ before expression, Lane 7. P_3_ after expression); (B) protein purification. Different fractions of purified P_1_, P_2_, and P_3_ proteins on SDS‐PAGE are shown. On the left is the molecular weight marker. (C) Protein expression confirmation by Western blotting. The blue bands are the expressed proteins. On the left is the molecular weight marker

The corresponding genes for P_1_, P_2_, and P_3_ vaccine candidates were optimized according to the *E*. *coli* codon usage bias, and the unwanted sequences, which could affect the optimal protein expression, were omitted. DNA sequencing and enzymatic digestion on the subcloned vector confirmed Gene cloning. The colony PCR confirmed that the vectors are transformed into the *E*. *coli* BL21 (DE3) host. The results of the protein expression have shown that P_1_, P_2_, and P_3_ proteins are overexpressed and can travel to the expected molecular weight on SDS‐PAGE gel (12 kDa for P_1_, 14 kDa for P_2_, and 25 kDa for P_3_) (Figure 1A). It has been revealed that the best condition for the expression of the proteins is adding 1mM of IPTG and shaking for 4 h at 37°C.

### 3.4 Protein purification and Western blot analysis

The expressed proteins for P_1_, P_2_, and P_3_ were purified by Ni^+^–NTA resin‐packed columns following the denaturation and renaturation procedure following dialysis (pH change and urea alternation). Running the purified protein samples on the SDS‐PAGE gel indicated that the unwanted proteins moieties were removed from the samples, and the purified proteins could travel to their expected molecular weight (Figure 1B). The Western blot analysis has also confirmed the identity of the purified antigens using the anti‐His tag antibody (Figure 1C).

### 3.5 CD analyses

The results of the CD analyses for the P_1_, P_2_, and P_3_ vaccine candidates were listed in Table 1, which indicated that all three vaccine candidates were folded to discernible secondary structures with different ratios.

**TABLE 1:** CD results for the P1, P2, and P3 vaccine candidates (0‐M urea is the base buffer without urea)

Secondary structure	PBS	0 M Urea	1 M Urea	2 M Urea	4 M Urea	6 M Urea	8 M Urea	Prediction (%)
P_1_								
α‐helix	12.10	100.00	35.80	48.00	23.30	0.00	32.30	5.94
β‐sheet	61.60	0.00	0.00	0.00	38.50	60.00	0.0	26.73
β‐turn	26.30	0.00	64.20	52.00	28.70	0.00	41.90	5.94
Random coil	0.00	0.00	0.00	0.00	9.50	40.00	25.80	61.39
P_2_								
α‐helix	36.00	33.90	49.70	12.80	13.30	85.50	56.40	38.14
β‐sheet	0.00	66.10	0.00	54.70	34.60	0.00	0.00	12.37
β‐turn	64.00	0.00	50.30	7.50	31.50	0.00	43.60	5.16
Random coil	0.00	0.00	0.00	25.00	20.60	14.50	0.00	44.33
P_3_								
α‐helix	7.70	74.70	38.00	75.10	19.80	100.0	59.20	14.37
β‐sheet	33.50	0.00	0.00	0.00	52.20	0.00	0.00	26.35
β‐turn	36.70	0.00	62.00	0.00	21.90	0.00	40.80	8.98
Random coil	22.00	25.30	0.00	24.90	6.10	0.00	0.00	50.30

### 3.6 Antibody production

**FIGURE 2:**
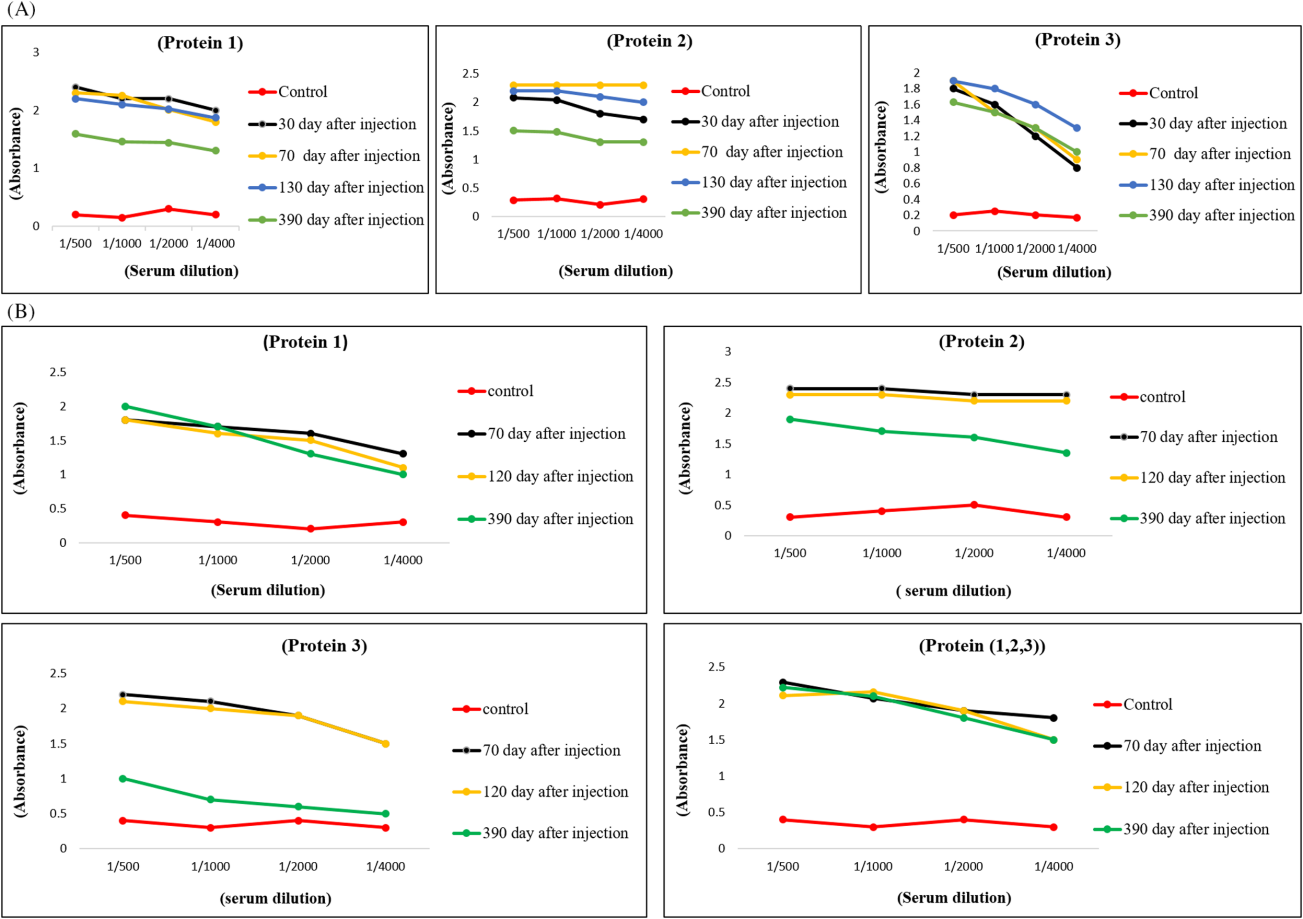
(A) Serum antibody titer for rabbits immunized with P_1_, P_2_, and P_3_ proteins compared to the control group in 30, 70, 130, and 390 days after injection. (B) The serum antibody titer for mice groups immunized with P_1_, P_2_, P_3_, and a mixture of three proteins compared to the control group in 70, 120, and 390 days after injection. All tests were performed duplicate

After performing the immunization regiment on both rabbits and mice groups, their serum samples were evaluated for antibody elicitation. The results of indirect ELISA tests for both animal groups indicated that antibodies were raised against P_1_, P_2_, and P_3_ protein candidates (Figure 2A,B). The results of these indirect ELISA tests confirmed the immunogenicity of the P_1_, P_2_, and P_3_ protein candidates within both rabbits and mice groups without any lethal consequences. The results have also confirmed long‐lasting antibody response for P_1_, P_2_, and P_3_ protein candidates and showed that the antibody titer for all three rabbit groups (injected with P_1_, P_2_, and P_3_ proteins) remains higher than the control group up to 390 days after injection. Similarly, the antibody titer was higher than the control group for mice groups (injected with P_3_, P_1_, P_2_, and a mixture of three proteins) up to 390 days after the second injection. However, a decreasing trend for antibody titer was detected in the test results after 390 days compared to that of the previous days.

### 3.7 ELISA on serum sample of COVID‐19 patients

**FIGURE 3:**
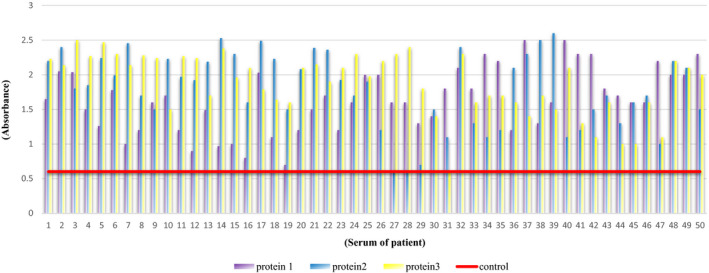
The ELISA results for anti‐SARS‐CoV‐2 antibody detection in the serum of COVID‐19 patients using the P_1_, P_2_, and P_3_ proteins as capture antigens compared to the healthy subject

Using the P_1_, P_2_, and P_3_ protein as the capture antigen in an ELISA may show the ability of these antigens to interact with antibodies produced within the serums of COVID‐19 patients. Our results indicated that P_1_, P_2_, and P_3_ vaccine candidates are capable of interacting with antibodies raised within the serums from COVID‐19 patients (Figure 3). A positive interaction result could be construed as functional and structural similarities between the P_1_, P_2_, and P_3_ vaccine candidates and their corresponding sequences within the whole virus structure.

### 3.8 Antibody purification and CPE‐based neutralization assays

**FIGURE 4:**
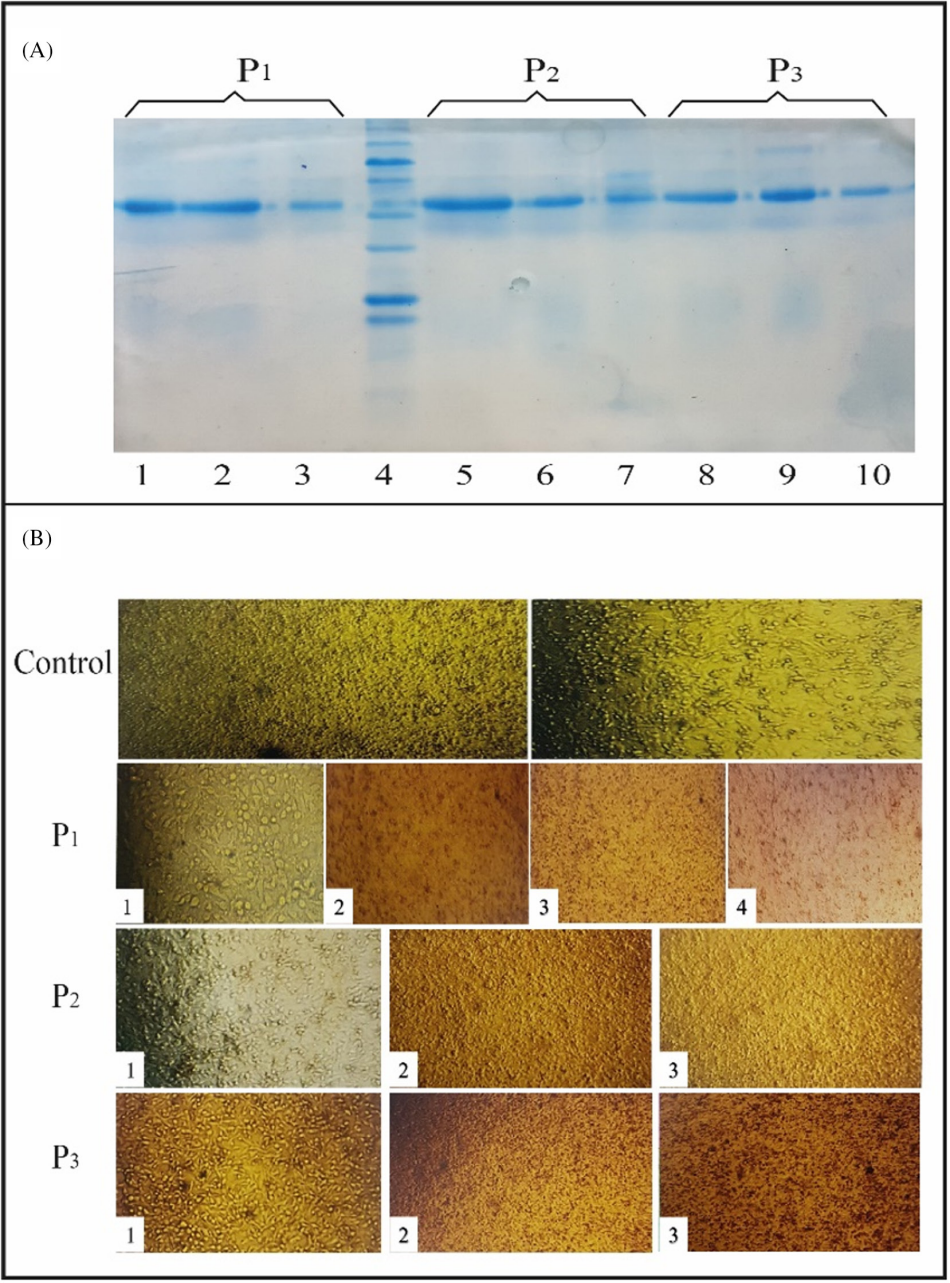
(A) Purification of antibody. The fractions of antibody elution from the protein A column (lanes 1–3: elusion fractions for P_1_, lane 4: molecular weight, lanes 5–7: elusion fractions for P_2_, lanes 8–10: elusion fractions for P_3_). (B) Different concentrations of virus neutralization via the antibodies produced by P_1_, P_2_, and P_3_ vaccine candidates (1: is the 1:10 serum and 1:10 virus dilution, 2: is the 1:10 serum and 1:100 virus dilution, 3: is the 1:10 serum and 1:1000 virus dilution, 4: is the 1:100 serum and 1:1000 virus dilution) compared with the control group

The affinity chromatography on a Protein A column managed to purify the IgG antibodies from the rabbit serum samples (Figure 4A). The neutralization assay determines the effect of neutralizing antibodies based on the observation of cell morphology. This method is reported to be the first and most frequently used neutralization assay in the SARS research.^33^ The results indicated that it takes 5 days for the infected Vero E6 cells to start forming visible CPE including dissociated cell patterns. It was evident that the 1:10 dilution of the rabbit antibody (equivalent to 50 µg) immunized by the P_1_, P_2_, and P_3_ vaccine candidates could neutralize the 1:1000, 1:100, and 1:10 dilution of viral stock. The 1:100 concentration of the rabbit serum immunized by P_1_ vaccine candidate could neutralize up to the 1:1000 concentrations of viral stock (Figure 4B).

## 4 DISCUSSION

Employing an integrative approach, we aimed to produce a safe and inexpensive vaccine against the SARS‐CoV‐2. In this regard, the subunit vaccine platform is based on S protein. Previous studies on SARS‐CoV, MERS‐CoV, and recent SARS‐CoV‐2 have revealed that given the critical role of S protein in the mechanism behind the virus entry into the host cells, it can be deemed as the best candidate for vaccine development efforts. Usually, the whole S protein (or its subunits) or the RBD region is used for the design of a subunit vaccines against the SARS‐CoV‐2. The vaccine is usually injected along with a suitable adjuvant to get the best immunization results. The Novavax subunit vaccine uses the complete S glycoprotein in combination with the MATRIX M adjuvant. This vaccine is now in phase 2 clinical trials. Previous studies have reported that selecting the whole sequence of the S protein could trigger unwanted immune responses. These responses could lead to inflammatory and hepatic damage or increased infection after exposure to SARS‐CoV in the animal models.^34^, ^35^, ^36^, ^37^ In light of these facts, encompassing the whole S protein sequence in the designed subunit vaccine could be associated with adverse effects.^34^, ^35^, ^36^, ^37^ Inclusion of the RBD region instead of the whole S protein is offered as an alternative for anti‐SARS‐CoV‐2 vaccine design efforts. This region has been shown to produce a higher antibody titer compared to the immunization with the whole S protein. The rational underlying this observation could be the existence of immuno‐dominant non‐neutralizing epitopes within the sequence of the whole S protein sequence. These epitopes would trigger the immune system toward themselves, and the neutralizing epitopes would remain unresponsive. Ultimately, the elicitation of sufficient titer of the neutralizing antibodies would not be accomplished.^31^, ^38^, ^39^, ^40^, ^41^ Monoclonal antibodies produced against different epitopes of the RBD region of S protein have been reported to be effective against different strains of the SARS virus isolated from patients at various stages of the disease. These antibodies are predicted to provoke neutralizing effects on SARS‐CoV‐2. Moreover, unlike inactivated vaccines based on whole viruses, RBD‐based immunization was not associated with antibody‐dependent enhancement (ADE) or other detrimental immune responses.^42^

In silico studies have garnered a lot of attention in the design of subunit vaccines. Kar et al have developed a multiepitope vaccine based on the selected epitopes from the S glycoprotein of the SARS‐CoV‐2.^43^ In a similar approach, another multiepitope vaccine was designed and assessed based on the S protein epitopes of the SARS‐CoV‐2.^44^ The promising results of these studies have convinced us to use a combination of in silico studies and information from previous studies to design three vaccine candidate antigens. The extent of conservancy, glycosylation, toxicity, antigenicity, and immunogenicity were the criteria considered for the vaccine design and selection.

Although the eukaryotic expression systems offer some advantages, they are still more costly, laborious, and time consuming.^45^ On the other hand, among 151 recombinant protein drugs, which are approved by FDA and European Medicines Agency, more than 45 drugs are *E*. *coli* derived.^46^ In the period from 2010 to 2014, 29% of the recombinant drugs were expressed within *E*. *coli*.^47^ It has also been reported that the vaccines based on the spike protein, which are expressed in *E*. *coli*, could induce specific blocking antibodies.^48^ Numerous other studies have also reported the merits of *E*. *coli*‐derived recombinant proteins. One of the most significant concerns about the exploitation of *E*. *coli* expression system is it inability to mimic the eukaryotic post‐translational modifications. However, we have avoided these regions during the vaccine design stage, which would circumvent the possible complications upon administration of the antigens. Folding to a native‐like structure is highly important for the designed recombinant proteins to invoke proper immune responses. Since the protein purification was performed in urea containing solutions, the secondary structure of the protein would be affected. Urea can denature the protein structures by direct and indirect mechanisms. Regarding the direct mechanism, the urea could bind directly to the charged and polar side chains of the protein via hydrogen bonding and other electrostatic interactions, it could bind directly to amino acids through van der Waals attractions, and it could bind the side chains via a combination of these two methods. Stronger dispersion interaction of the urea with protein than water supports the direct interaction mechanism.^49^ This is while in the indirect mechanism, urea leads to the easier dissolution of hydrophobic protein groups by disrupting the structure of water. It has been reported that with a gradual increase in urea concentration from 6 to 10 M, the beta sheets will be destroyed, the alpha helixes will remain stable and may even increase, and the random coils will remain intact.^50^, ^51^ The obtained CD results for the P_1_, P_2_, and P_3_ vaccine candidates have confirmed these structural changes during urea removal. This could be construed as protein refolding to its native structure upon urea removal.

Yuxian et al.^52^ have also used a recombinant fusion protein (RBD‐Fc) as an immunogen to immunize rabbits. The RBD region was selected due to its functional role in virus entry and inclusion of neutralizing epitope. The RBD‐Fc antigen induced robust antibody responses and ultimately prevented the SARS‐CoV infection in the diluted serum.^31^ Similar results were reported in a study conducted by Du et al.^53^ in mice. In line with the results of previous studies, our results have shown that the injection of purified proteins of different parts of S protein has led to a significant increase in the virus‐neutralizing antibody of the immunized animals. This antibody remained high for 390 days after the initial injections for rabbit and mouse groups. This property indicates the elicitation of a robust immune response. The ELISA test against the serums of the COVID‐19 patients confirmed the ability of the elicited polyclonal response to detecting the S protein within the serum of COVID‐19 patients.

To have an efficient immune response against the SARS‐CoV‐2, the elicited antibodies should exhibit the ability to neutralize the viral particle. The Virus Neutralization Test (VNT) method is a susceptible and specific test to check for the presence of neutralizing antibodies against the target virus. This method is also practiced as the gold standard method to analyze the presence of neutralizing antibodies against SARS‐CoV‐2. Previous studies have demonstrated that the S protein components such as S2, S1, and especially RBD hold a profound potential for production of neutralizing antibodies. These antibodies can block the virus binding to the ACE2 receptor and its membrane fusion.^31^ Our VNT results have also confirmed that the elicited antibodies against the designed proteins are highly persistent and capable of neutralizing the cultured live virus. This property could be construed as the highly promising potential of these proteins as vaccine candidates. However, for further analyses, these recombinant proteins should be injected into a higher mammal like Macaque rhesus monkey to assess their efficiency in higher species. Currently, we are conducting this experiment on the Macaque rhesus monkey. The available data (although auspicious) are not sufficient for definitive conclusions, and more time and experiments are needed to show the performance of such recombinant proteins in higher species. However, considering the results, these antigens appear to be compelling vaccine candidates against the SARS‐CoV‐2 owing to their ability to develop potent neutralizing antibodies, long‐term immunity in animals, and no apparent side effects. Future studies will disclose the true potential of these subunit vaccine candidates for vaccination against COVID‐19.

## Data Availability

Data are available on request.
